# *Chlamydia felis* exposure in companion dogs and cats in Lanzhou, China: a public health concern

**DOI:** 10.1186/1746-6148-9-104

**Published:** 2013-05-21

**Authors:** Song-Ming Wu, Si-Yang Huang, Min-Jun Xu, Dong-Hui Zhou, Hui-Qun Song, Xing-Quan Zhu

**Affiliations:** 1 State Key Laboratory of Veterinary Etiological Biology, Lanzhou Veterinary Research Institute, Chinese Academy of Agricultural Sciences, Lanzhou, Gansu Province 730046, PR China; 2 College of Animal Science and Veterinary Medicine, Heilongjiang Bayi Agricultural University, Daqing, Heilongjiang Province 163319, PR China

**Keywords:** *Chlamydia felis*, Chlamydiosis, Survey, Indirect hemagglutination (IHA) test, Cats, Dogs

## Abstract

**Background:**

Chlamydiaceae is a family of obligate intracellular pathogens with a worldwide distribution in many animal species, including humans. No information exists on the prevalence of *Chlamydia felis* infections in cats and dogs in Lanzhou, the geographical center of China. The aim of this study was to carry out a census of cats and dogs in Lanzhou and document the seroprevalence of *C. felis* exposure in these companion animals.

**Results:**

In this study, blood samples were collected from 485 animals (221 cats and 264 pet dogs) in Lanzhou between November 2010 and July 2011 to identify antibodies against *C. felis*. Thirteen of 221 (5.9%) cats and 32 of 264 (12.1%) pet dogs were positive for *C. felis* infection using indirect hemagglutination at a cutoff of 1:16. The seroprevalence in household and stray cats was 3.9% and 14.3%, respectively, and the difference was statistically significant (*P <* 0.05). Among different age groups, the seroprevalence in cats varied from 1.9 to 7.9%, and that in dogs ranged from 9.6 to 20.4%; however, the differences were not statistically significant (*P >* 0.05).

**Conclusions:**

This is the first report of the seroprevalence of *C. felis* exposure in cats and dogs in Lanzhou, northwestern China. Our results indicate that the presence of *C. felis* exposure in cats and dogs may pose a potential threat to human health.

## Background

*Chlamydia* is a genus comprising important zoonotic obligate intracellular pathogens that affect humans and a wide range of animals, including birds [1,2]. *Chlamydia* infection causes a wide spectrum of diseases in nonhuman mammals and birds, including atypical pneumonia, enteritis, conjunctivitis, endocarditis, and even abortion, resulting in heavy economic losses [3-6]. Several *Chlamydia* species are transmissible to humans and are of serious public health significance because they may lead to pneumonia, atherosclerosis, coronary heart disease, and other severe diseases. *Chlamydia abortus* and *C. psittaci* are of particular importance because they can cause abortion and psittacosis, respectively, in animals, birds and humans. The animal diseases caused by these microorganisms should be given more attention in terms of their zoonotic aspects and the zoonotic potential of other animal pathogens [2,7-9].

*Chlamydia felis* is an important agent with zoonotic potential. It causes primary infections in the upper respiratory tract and eyes of cats. It is usually transmitted through the air and in secretions from infected cats’ eyes or noses [10]. Clinical signs in dogs are similar to those associated with canine distemper, such as conjunctivitis, encephalitis, pneumonia, and keratitis [11,12]. Pet cats and dogs are considered to be faithful friends and companions of humans, thus playing an important role in human life. Unfortunately, cats and dogs may be important sources of *C. felis* infection in humans [8].

A study in China reported that severe pneumonia in peacocks and peacock farmers was caused by *C. psittaci*[13]. Another study indicated that 95 of 455 (20.9%) Tibetan sheep were seropositive for *C. abortus* antibodies at the cutoff of 1:16 [14], and the seroprevalence of *Chlamydia* infection in Tibetan pigs in Tibet was 16.63% between April and December 2010 [15]. Zhou *et al.* reported that the overall seroprevalence of chlamydial infection in dairy cattle was 7.25% in Guangzhou [16]. Xu *et al.* reported that 30.78% of pigs were positive for *Chlamydia* in Guangdong Province between March 2008 and May 2009 [12]. Surveys of *C. felis* infection in cats have been extensively reported worldwide [10], but limited information on *C. felis* infection in dogs is available. The present study was undertaken to characterize *C. felis* exposure and measure its seroprevalence in cats and pet dogs in Lanzhou, northwest China.

## Methods

The cats and pet dogs examined in the present study were handled in accordance with the Good Animal Practice requirements of the Animal Ethics Procedures and Guidelines of the People’s Republic of China. This study was approved by the Animal Ethics Committee of Lanzhou Veterinary Research Institute, Chinese Academy of Agricultural Sciences (Approval No. LVRIAEC2010-010). Informed client consent was obtained from the owners of all cats and pet dogs, and the study adhered to the highest standard (best practice) of veterinary care.

### Cat and dog serum samples

Blood samples were collected from randomly selected cats and dogs between November 2010 and July 2011 in Lanzhou, northwest China. These cats and pet dogs were admitted to pet hospitals located in four districts of Lanzhou (Chengguan, Anning, Xigu and Qilihe districts). Some required blood sample examination, and half of these blood samples were randomly selected for this study because we could not evaluate all of them. The examined pets were admitted to the pet hospitals for various diseases and health problems; the details of each animal’s disease condition were not available. Pet owners were asked about the breed, age, gender and geographical origin, and the biometric data of stray cats were estimated based on body condition and dental age. Blood samples were slowly collected from either the jugular or cephalic vein into labeled plain red-top tubes. The samples were transported to the laboratory and kept at room temperature for 2 h to allow for clot formation. The blood-filled tubes were then centrifuged at 1500 *g* or 3000 rpm for 5 to 10 min. After centrifugation, it was ensured that the gel had formed a clear barrier between the cells and the serum. The serum samples were separated and stored at −20°C until further analysis.

### Detection reagents

Antibodies to *C. felis* in cats and dogs were measured using a commercially marketed indirect hemagglutination (IHA) kit purchased from the Lanzhou Veterinary Research Institute of the Chinese Academy of Agricultural Sciences. The sensitivity and specificity values for the testing kit used in this study have been validated by ministry of agriculture of the People’s Republic of China (NY/T 562–2002).

### Detection procedure

The detection procedure followed the manufacturer’s instructions as previously described [12,14-17]. Briefly, sera were added to a 96-well V-bottomed polystyrene plate and diluted two-fold up to 1:2048, starting at 1:4. The detected antigen was added to each well, and the plate was then shaken slightly for 2 min followed by incubation at 37°C for 2 h. IHA titers of 1:16 or higher with the formation of an agglutinated erythrocyte layer were considered to be positive [12,14-17]; sera with dubious results were retested. According to the manufacturer’s instructions, 1:16 is the normal cutoff point for detecting a positive sample. If we had chosen 1:8, the false-positive rate would have been higher; if 1:32 had been chosen, the true-negative rate would have been lower. Positive (sera from infected dogs) and negative (sera from negative dogs) controls provided by the manufacturer were included in each test and assayed at the same dilutions of the sera samples.

### Statistical analysis

We evaluated the univariate effects of gender, age, and types of cats on the presence of antibodies against *C. felis* with a Chi square test by the SPSS for Windows (Release 18.0 standard version, SPSS Inc., Chicago, Illinois). The differences were considered statistically significant when *P* < 0.05. The exact binomial confidence interval for each group was calculated.

## Results

In this study, serum samples were collected from a total of 221 cats (179 household cats and 42 strays) and 264 pet dogs in Lanzhou, northwest China, and the antibodies to *C. felis* were measured by the IHA test. Thirteen of 221 (5.9%) examined cats were seropositive for *C. felis* infection, and 32 of 264 (12.1%) pet dogs were found to be positive for *C. felis* infection at the cutoff 1:16 (Tables 1 and 2). Among different age groups, the seroprevalence in cats varied from 1.9 to 7.9%, and that in dogs ranged from 9.6 to 20.4% (Tables 1 and 2). The seroprevalence in household and stray cats was 3.9 and 14.3%, respectively; however, dogs were only grouped in one category (household pets). The samples were collected in four different animal hospitals in the same city. Because the city is not large, comparative analyses were not performed.

**Table 1:** **Seroprevalence of *Chlamydia felis *infection in household and stray cats by gender and age in Lanzhou, northwest China using indirect haemagglutination**

**Cat groups**	**Types of cat**			**95% CI**				**95% CI**	**Total**			**95% CI**
	**Household cats**				**Stray cats**							
	**No. tested**	**No. positive**	**Prevalence (%)**		**No. tested**	**No. positive**	**Prevalence (%)**		**No. tested**	**No. positive**	**Prevalence (%)**	
Gender												
Male	87	4	4.6	1.26-10.99	17	1	5.9	0.15-28.89	104	5	4.8	1.59-10.52
Female	92	3	3.3	0.68-8.96	25	5	20	6.81-40.72	117	8	6.8	3.04-12.37
Age (years)												
<1	41	0	0		12	1	8.3	0.21-38.47	53	1	1.9	0.04-10.00
1 ≤ Yr < 2	65	2	3.1	0.37-10.71	24	5	20.8	7.12-41.96	89	7	7.9	3.28-15.15
2 ≤ Yr < 3	37	2	5.4	0.66-18.39	3	0	0		40	2	5.0	0.61-17.19
≥3	36	3	8.3	1.75-22.61	3	0	0		39	3	7.7	1.61-21.12
Total	179	7	3.9	1.60-7.84	42	6	14.3	5.38-28.59	221	13	5.9	3.20-9.56

**Table 2:** **Prevalence of antibodies to *Chlamydia felis *in pet dogs by gender and age in Lanzhou, northwest China using indirect haemagglutination**

**Biometric data**	**No. tested**	**No. positive**	**Prevalence (%)**	**95% CI**
Gender				
Male	139	14	10.1	5.72-15.85
Female	125	18	14.4	9.22-20.81
Age (years)				
<1	83	8	9.6	4.34-17.02
1 ≤ Yr < 2	64	5	7.8	2.58-17.25
2 ≤ Yr < 3	49	10	20.4	10.12-35.36
≥3	68	9	13.2	6.22-23.57
Total	264	32	12.1	8.49-16.51

## Discussion

Epidemiological data regarding the distribution of animal chlamydiosis in China are scarce, and most of them are on food animals such as sheep, cattle, and pigs [14-16]. Companion animals, such as pet cats and dogs, are considered to be faithful friends of humans; however, cats and dogs could be important sources of *Chlamydia* infection in humans. Therefore, the present study aimed to estimate the chlamydial seroprevalence in cats and pet dogs in Lanzhou, northwest China. Several studies have reported the *C. felis* prevalence in cats in various regions. For example, Halánová M *et al.*[18] used direct immunofluorescence and detected an overall 45.16% prevalence of *C. felis* among cats in Slovak, Millán, and Rodríguez [19] detected a 27% seroprevalence in serum samples from European wildcats using enzyme-linked immunosorbent assay. The overall seroprevalence of *C. felis* exposure in cats in Lanzhou was 5.9%, which is lower than those in the above mentioned studies but higher than that observed in cats in Dongguan (2.38%), southern China, using the same commercial IHA kit [20]. The differences in the seroprevalence of *C. felis* exposure in cats could be related to differences in ecological and geographical factors such as temperature, rainfall, or landscape differences; serologic tests used; feeding; and animal welfare protocols for cats.

Our results indicate that the seroprevalence of *C. felis* exposure in stray cats (14.3%) was significantly higher than that observed in household cats (3.9%) (*P <* 0.05), which supports the results obtained by Halánová [18]. These differences are probably attributed to lifestyle and animal welfare protocols. Stray cats have more opportunities to come into contact with infected birds or other animals, and they suffer from poor nutrition and possibly compromised immune systems that may contribute to increased exposure to infectious pathogens. This may explain the higher *C. felis* prevalence in stray cats than in pet cats. One study reported that a large number of cats and dogs become unowned each year in the UK, which may have considerable implications for their welfare [21].

Studies using both culture and polymerase chain reaction (PCR) indicated that cats less than 1 year of age were the most likely to be infected with *Chlamydia*, and cats older than 5 years were the least likely to be infected [10]. However, although the differences were not statistically significant, we found a higher prevalence of antibodies to *C. felis* in older animals in this study. The *C. felis* seroprevalence in male and female cats were different, but statistical analysis showed no significant difference. This suggests that *C. felis* exposure in the cats in this study had no sex predilection, consistent with previous studies.

In addition to in cats, a high prevalence of *Chlamydia* has been found in growing pigs with or without clinical diseases [22], and chlamydial infections occur frequently in German sheep flocks, even in the absence of elevated abortion rates [23]. Reports of canine chlamydiosis are not common, possibly because *C. felis* is rarely considered to be a disease-causing pathogen in dogs. However, a few studies found chlamydial infections in clinically normal dogs [24,25]. For example, Pantchev *et al.* used species-specific real-time PCR assays and revealed that four of five dogs were infected by *C. felis*. The data of that study suggested that this species is highly adapted to cats and that despite the high prevalence of infection, *C. felis*-positive dogs, unlike cats, seldom suffer from conjunctivitis [25].

There is limited knowledge about chlamydiosis in dogs in China. Hence, we reported the prevalence of *C. felis* exposure in pet dogs in northwest China for the first time. In the present study, antibodies to *C. felis* were found in 32 (12.1%) of 264 pet dogs, which is a higher prevalence than that detected in Dongguan (2.87%), southern China [20]. Compared with other age groups, a higher prevalence of *C. felis* exposure was found in 2-year-old cats; however, the difference was not statistically significant among different age groups (*P >* 0.05).

The results of this study revealed that *C. felis* is highly prevalent among the cats and pet dogs in Lanzhou, China, which suggests that this pathogen could be a significant cause of ocular diseases in these animals. *Chlamydia felis* is zoonotic pathogen, and maintenance of hygienic conditions and prompt treatment of affected cats and dogs is recommended to prevent human disease. The samples in the present study were collected between November 2010 and July 2011, which is a short period of time; thus, the obtained result may not reflect the actual situation of *C. felis* infection during longer periods. However, our results provide useful information for future studies. A number of factors may contribute to the varying seroprevalence, such as geographical conditions, diagnostic methods, feeding and living styles, and stress. In future studies, it would be interesting to test environmental samples such as soil, water, air, and ventilation systems to assess the contamination level of premises with *C. felis*.

## Conclusion

The results of this investigation indicate the presence of *C. felis* infection in cats and pet dogs in northwestern China, which raises a public health concern because dogs and cats could be reservoir hosts of *C. felis* infection in humans. To our knowledge, this is the first report of *C. felis* infection in cats and dogs in northwest China.

## Competing interests

The authors declare that they have no competing interests.

## Authors’ contributions

XQZ and SYH conceived and designed the study and wrote and critically revised the manuscript. SMW, SYH, and MJX performed the experiments, analyzed the data, and drafted the manuscript. DHZ and HQS helped in the study design, study implementation, and manuscript revision. All authors read and approved the final manuscript.
